# Delay in diagnosis of influenza A (H1N1)pdm09 virus infection in critically ill patients and impact on clinical outcome

**DOI:** 10.1186/s13054-016-1512-1

**Published:** 2016-10-23

**Authors:** Francisco Álvarez-Lerma, Judith Marín-Corral, Clara Vila, Joan Ramón Masclans, Francisco Javier González de Molina, Ignacio Martín Loeches, Sandra Barbadillo, Alejandro Rodríguez, on behalf of the H1N1 GETGAG/SEMICYUC Study Group, Pedro Cobo, Javier Martins, Cecilia Carbayo, Emilio Robles-Musso, Antonio Cárdenas, Javier Fierro, Dolores Ocaña Fernández, Rafael Sierra, Ma Jesús Huertos, Ma Luz Carmona Pérez, Juan Carlos Pozo Laderas, R. Guerrero, Juan Carlos Robles, Melissa Echevarría León, Alberto Bermejo Gómez, Enrique Márquez, Manuel Rodríguez-Carvajal, Ángel Estella, José Pomares, José Luis Ballesteros, Olga Moreno Romero, Yolanda Fernández, Francisco Lobato, José F. Prieto, José Albofedo-Sánchez, Pilar Martínez, María Victoria de la Torre, María Nieto, Estefanía Cámara Sola, Miguel Ángel Díaz Castellanos, Guillermo Sevilla Soler, Carlos Ortiz Leyba, José Garnacho-Montero, Rafael Hinojosa, Esteban Fernández, Ana Loza, Cristóbal León, Samuel González López, Ángel Arenzana, Dolores Ocaña, Inés Navarrete, Medhi Zaheri Beryanaki, Ignacio Sánchez, Manuel Pérez Alé, Ana Ma Poullet Brea, Juan Francisco Machado Casas, Carlos Serón, Manuel Luis Avellanas, Arantxa Lander, S. Garrido Ramírez de Arellano, M. I. Marquina Lacueva, Pilar Luque, Elena Plumed Serrano, Juan Francisco Martín Lázaro, Carlos Sánchez Polo, Isabel Gutiérrez Cía, Belén Jiménez Bartolomé, Carlos López Núñez, Ignacio González, José Ignacio Tomás Marsilla, Clara Jaques Andrés, Pablo Gutiérrez Ibañes, Pilar Araujo Aguilar, Jose Ma Montón, Paloma Dorado Regil, Lisardo Iglesias, Carmen Pascual González, Brígida Quindós Fernández, Lorena Martín Iglesias, Lucía Viña Soria, Raquel Yano Escudero, Ma del Rosario Martínez Revuelta, José Ma Quiroga Ruiz, Águeda García-Rodríguez, Marta Martín Cuadrado, Ana Luz Balán Mariño, Lorenzo Socias, Pedro Ibáñez, Marcío Borges-Sa, A. Socias, A. Del Castillo, Ricard Jordà Marcos, Cristina Muñoz, José M. Bonell Goytisolo, José Antonio Morales Carbonero, Ignacio Ayestarán, M. Ángeles González López, Cecilia Vilanova Pàmies, Rossana Pérez Senoff, Marta Generelo López de Medrano, Sergio Ruiz-Santana, Juan José Díaz, Catalina Sánchez Ramírez, Montse Sisón, David Hernández, Ana Trujillo, Luis Regalado, Sonia Rodríguez Fernández, Leonardo Lorente, Judith Cabrera Rivero, Ma Luisa Mora Quintero, Mar Martín, Sergio Martínez, J. J. Cáceres, Manuel Sánchez Palacio, D. García Rodríguez, María Ripoll Leria, Borja Suberviola, P. Ugarte, Fernando García-López, Rafael Sánchez Iniesta, Ángel Álvaro Alonso, Antonio Padilla, Basi Martínez Palacios, Ma Luisa Gómez Grande, Ma Carmen Martín Rodríguez, Hasania Adbel-Hadi Álvarez, Alfonso Ambros Checa, Higinio Martín Hernández, Antonio Albaya, Alberto Silva Obregón, Carlos Marian Crespo, Carlos Armendáriz Estrella, Carmen Benito Puncel, Eduardo Quirós Oyargue, Alfonso Canabal, Luis Marina, Ismael López de Toro, Almudena Simón, José María Añón, Ma Jesús López Pueyo, María del Valle Ortiz, Sergio Ossa Echeverri, Zulema Ferreras, Juan C. Ballesteros Herraez, Santiago Macías, José Ángel Berezo, Jesús Blanco Varela, Pablo Blanco Schweizer, Ángela González Salamanca, Luis Tamayo Lomas, Andaluz Ojeda Anzález, Ramón Cicuéndez Ávila, G. Francisco Javier Pérez, Antonio Álvarez Terrero, Fabiola Tena Ezpeleta, Christian Sala, Oliverio López, Zulema Páez, Álvaro García, Demetrio Carriedo Ule, Miriam Riesco Crespo, Jesús Pino Rebolledo, Nicolás Hidalgo Andrés, Ana Carolina Caballero Zirena, Belén Román García, Juan Bautista López Messa, Rosa Ma Catalán, Miquel Ferrer, Antoni Torres, Catia Cilloniz, Sandra Barbadillo Ansorregui, Lluís Cabré, Ignacio Baeza, Assumpta Rovira, Francisco Álvarez-Lerma, Antonia Vázquez, Joan Nolla, Francisco Fernández, Joaquim Ramón Cervelló, Raquel Iglesia, Rafael Mañéz, J. Ballús, Rosa Ma Granada, Jordi Vallés, Emili Díaz, Marta Ortíz, C. Guía, Ignacio Martín-Loeches, Joaquim Páez, Jordi Almirall, Xavier Balanzó, Estel Güell, Juan Carlos Yébenes, Jordi Rello, Elena Arnau, Marcos Pérez, César Laborda, Jesica Souto, Leonel Lagunes, Iñaki Catalán, Josep Ma Sirvent, Nerea López de Arbina, Anna Baró Serra, Adriana Sánchez, Silvia M. Cuenca, Mariona Badía, Begonia Baseda-Garrido, Montserrat Valverdú-Vidal, Fernando Barcenilla, Mercedes Palomar, Xavier Nuvials, Pedro Garrido Benedicto, Ferrán Roche Campo, M. F. Esteban, José Luna, Gaspar Masdeu Eixarch, Angels Pascual Diago, Juan Ma Nava, J. González de Molina, Josep Trenado, Ricard Ferrer, Zoran Josic, Montserrat Casanovas, Francisco Gurri, Paula Rodríguez, Alejandro Rodríguez, Laura Claverías, Sandra Trefler, María Bodí, Mónica Magret, Cristina Ferri, Rosa María Díaz, Eduard Mesalles, Fernando Arméstar, Diego de Mendoza, Carmen Lomas Fernández, José Julián Berrade, Alfonso Bonet Saris, Marina Pechkova, Cristina Mora Jiménez, Santiago Picos Gil, Juliá-Narváez José, Manuel Robles Marcos, Vanessa Farje Mallqui, Ma Ángeles Santiago Triviño, Pablo Martínez García, Alberto Fernández-Zapata, Teresa Recio, Abilio Arrascaeta, Ma José García-Ramos, Elena Gallego, Esther Saiz Rodrigo, Fernando Bueno, Mercedes Díaz, Noemí Gil Pérez, David López Hormigo, Juan Diego Jiménez Delgado, Pérez Frutos, M. J. Rivera Pinna, Ma Lourdes Cordero, José A. Pastor, Luis Álvarez-Rocha, Alexandra Ceniceros Barros, Alejandra Virgós Pedreira, Dolores Vila, Carmen Fernández González, Eleuterio Merayo, Víctor José López-Ciudad, Juan Cortés Cañones, Eva Vilaboy, José Villar Chao, Francisco Savira Cid López, Pablo Vidal Cortés, Marcos A. Pérez Veloso, Eva María Saborido, Enrique Alemparte Pardavila, Ana Ortega Montes, Raúl José González, Santiago Freita, Enrique Alemparte, Ana Ortega, Ana María López, Julio Canabal, Enrique Ferres, Javier Blanco Pérez, M. Ortiz Piquer, Santiago Freitas Ramos, Lucas Lage Cendón, Vanesa Gómez Casal, Sabela Vara Adrio, Eva Menor Fernández, Susana González Prado, Antonio Varela Franco, José Luis Monzón, Félix Goñi, Frutos Del Nogal Sáez, M. Blasco Navalpotro, Ricardo Díaz Abad, José Luis Flordelis Lasierra, Ma Carmen García-Torrejón, César Pérez-Calvo, Diego López, Luis Arnaiz, S. Sánchez-Alonso, Carlos Velayos, Francisco del Río, Miguel Ángel González, Mercedes Nieto, Carmen Sánchez Cesteros, María Cruz Martín, José Ma Molina, Juan Carlos Montejo, Mercedes Catalán, Patricia Albert, Ana de Pablo, José Eugenio Guerrero, María Zurita, Jaime Benítez Peyrat, Miriam Díaz Cámara, Enrique Cerdá, Manuel Alvarez, Carlos Pey, Eva Manteiga Riestra, Concepción Martínez-Fidalgo, Montse Rodríguez, Eduardo Palencia, Rafael Caballero, Concepción Vaquero, Francisco Mariscal, S. García, Rico Cepeda, Nieves Carrasco, Isidro Prieto, A. Liétor, R. Ramos, Rosario Cuadra Casas, Cruz Soriano Cuesta, Susana Sánchez Alonso, Beatriz Galván, Juan C. Figueira, M. Cruz Soriano, Belen Civantos Martín, Alejandro Robles Caballero, P. Galdós, Bárbara Balandín Moreno, Sara Alcántara Carmona, Fernández del Cabo, Cecilia Hermosa, Federico Gordo, Alejandro Algora, Amparo Paredes, Teodoro Grau Carmona, J. A. Cambronero, Esther López Ramos, Yaiza Ortiz de Zárate, Sonia Gómez-Rosado, Margarita Mas Lodo, Nieves Franco Garrobo, Silvia Álvarez Hernández, Teresa Honrubia, Luis Miguel Prado López, Esteban A. Lorente, J. A. Nin, Carlos Jaramillo Sotomayor, Luis Arnaiz, Esperanza Molero Silvero, Eduardo Morales Fernández de la Reguera, Rosa Ma de la Casa Monje, Fátima Martín Serrano, Ma Victoria Trasmonte Martínez, M. Cruz Martín Delgado, Sofía Martínez, F. Felices Abad, Isabel Cremades Navalon, Martín Vigil Velis, Mariano Martínez, Domingo Martínez Baño, Enriqueta Andreu, Sergio Manuel Butí, Bernardo Gil Rueda, Francisco García, Noemí Llamas Fernández, Luis Herrera Para, Alejandro Ortín Freire, Ma Rosa Navarro Ruiz, C. R. Hernández Romero, Enrique Maraví-Poma, I. Jimenez Urra, Laura Macaya Redin, A. Tellería, Josu Insausti, Noelia Artesero García, Laura Macaya, Joaquín Lobo Palanco, Nagore González, Pilar Marco, Loreto Vidaur, Estibaliz Salas, Ruth Salaberría Udabe, B. Santamaría, Tomás Rodríguez, Juan Carlos Vergara, Jose Ramón Iruretagoyena Amiano, Iratí Garrido Santos, Alberto Manzano, Carlos Castillo Arenal, Pedro María Olaechea, Higinio Martín Hernández, Alejandro Martín López, Fernando Fonseca San Miguel, José Blanquer, Nieves Carbonell, José Ferreres Franco, Roberto Reig Valero, A. Belenger, Susana Altaba, Bernabé Álvarez-Sánchez, José Cánovas Robles, Jaime Sánchez Francisco, Mar Ruiz Sánchez, Santiago Alberto Picos, Abilio Arrascaeta Llanes, Eugenio Herrero Gutiérrez, Alberto Fernández Zapata, Ángel Sánchez-Miralles, José Luis Antón Pascual, Juan Bonastre, M. Palamo, Javier Cebrián, José Cuñat, Mónica Gordón Sahuquillo, Belén Romero, Santiago Borrás Pallé, Javier de León Belmar, Rafael Zaragoza, Constantino Tormo, Susana Sancho Chinesta, Virgilio Paricio, Asunción Marqués, S. Sánchez-Morcillo, S. Tormo, J. Latour, M. Ángel García, Manuel Palomo, Francisco Tarín Royo, Pedro Manzano Hinojosa, Ma Salomé Sánchez Pino, Concha Maragues Ribes, Rubén González Luis, Antolí Ribas

**Affiliations:** 1 Service of Intensive Care Medicine, Hospital del Mar, Passeig Marítim 25-29, E-08003 Barcelona, Spain; 2 Research Group in Critical Disorders (GREPAC), Institut Hospital del Mar d’Investigacions Mèdiques (IMIM), Barcelona, Spain; 3 Universitat Autónoma de Barcelona, Barcelona, Spain; 4 CIBER de Enfermedades Respiratorias (CIBERES), Madrid, Spain; 5 Universitat Pompeu Fabra, Barcelona, Spain; 6 Service of Intensive Care Medicine, Hospital Universitari Mútua de Terrassa, Terrassa, Barcelona Spain; 7 Service of Intensive Care Medicine, St James Hospital, Dublin, Ireland; 8 Service of Intensive Care Medicine, Hospital General de Catalunya, Sant Cugat del Vallés, Barcelona, Spain; 9 Service of Intensive Care Medicine, Hospital Universitari Joan XXIII, IISPV-URV, Tarragona, Spain

**Keywords:** Influenza A (H1N1)pdm09 virus infection, Mortality, Critically ill, Early diagnosis, Late diagnosis, Outcome, ICU

## Abstract

**Background:**

Patients infected with influenza A (H1N1)pdm09 virus requiring admission to the ICU remain an important source of mortality during the influenza season. The objective of the study was to assess the impact of a delay in diagnosis of community-acquired influenza A (H1N1)pdm09 virus infection on clinical outcome in critically ill patients admitted to the ICU.

**Methods:**

A prospective multicenter observational cohort study was based on data from the GETGAG/SEMICYUC registry (2009–2015) collected by 148 Spanish ICUs. All patients admitted to the ICU in which diagnosis of influenza A (H1N1)pdm09 virus infection had been established within the first week of hospitalization were included. Patients were classified into two groups according to the time at which the diagnosis was made: early (within the first 2 days of hospital admission) and late (between the 3rd and 7th day of hospital admission). Factors associated with a delay in diagnosis were assessed by logistic regression analysis.

**Results:**

In 2059 ICU patients diagnosed with influenza A (H1N1)pdm09 virus infection within the first 7 days of hospitalization, the diagnosis was established early in 1314 (63.8 %) patients and late in the remaining 745 (36.2 %). Independent variables related to a late diagnosis were: age (odds ratio (OR) = 1.02, 95 % confidence interval (CI) 1.01–1.03, *P* < 0.001); first seasonal period (2009–2012) (OR = 2.08, 95 % CI 1.64–2.63, *P* < 0.001); days of hospital stay before ICU admission (OR = 1.26, 95 % CI 1.17–1.35, *P* < 0.001); mechanical ventilation (OR = 1.58, 95 % CI 1.17–2.13, *P* = 0.002); and continuous venovenous hemofiltration (OR = 1.54, 95 % CI 1.08–2.18, *P* = 0.016). The intra-ICU mortality was significantly higher among patients with late diagnosis as compared with early diagnosis (26.9 % vs 17.1 %, *P* < 0.001). Diagnostic delay was one independent risk factor for mortality (OR = 1.36, 95 % CI 1.03–1.81, *P* < 0.001).

**Conclusions:**

Late diagnosis of community-acquired influenza A (H1N1)pdm09 virus infection is associated with a delay in ICU admission, greater possibilities of respiratory and renal failure, and higher mortality rate. Delay in diagnosis of flu is an independent variable related to death.

## Background

Since the 2009 H1N1 influenza pandemic, patients with influenza A (H1N1)pdm09 admitted to the ICU remain an important source of mortality during the influenza season [1, 2]. The importance of early diagnosis and prompt start of antimicrobial treatment has been shown consistently in critically ill patients with severe bacterial infection or severe sepsis [3–6]. In patients with influenza A, in most cases typically during epidemic periods, antiviral treatment is administered when diagnosis is suspected (within the first 48 hours of hospital admission), although diagnosis and treatment (between the 3rd and 7th day of admission) can be delayed because of the lack of clinical suspicion by the medical team or negative results in the first samples analyzed (false negatives) [7, 8].

Different studies have identified factors independently associated with mortality in patients diagnosed with influenza A (H1N1)pdm09 infection [9, 10] or in selected subgroups, such as patients older than 65 years of age [11], obesity [12], immunodeficiency viral infection (HIV) [13], chronic liver disease [14], childhood [15] and pregnancy [16], as well as in different presenting forms of infection (severe sepsis, septic shock, pneumonia) and ICU admission [17, 18]. Also, other subsets of patients have been independently analyzed according to the presence of some factors, such as previous influenza vaccination [19], earliness of treatment with oseltamivir [20], use of corticoids [21] or macrolides [22], or the need for invasive or noninvasive mechanical ventilation on ICU admission or during the ICU stay [23]. However, the clinical impact of a delay in the diagnosis of influenza A (H1N1)pdm09virus infection is unknown, particularly in those patients ultimately requiring admission to the ICU.

The objective of the study was to analyze data available in a multicenter database of patients admitted to the ICU diagnosed with influenza A (H1N1)pdm09 virus infection, to determine clinical factors related to a delay in diagnosis and the impact on the outcome of patients. It was hypothesized that a delay in diagnosing influenza A (H1N1)pdm09 infection is associated with a worse clinical course and that early identification of influenza-infected patients can contribute to optimization of treatment.

## Methods

### Design and study population

This was a prospective, multicenter, observational cohort study. Between January 1, 2009 and December 31, 2015, data for all patients with microbiologically-confirmed diagnosis of influenza A (H1N1)pdm09 virus infection admitted to 148 ICUs throughout Spain were included in the GETGAG/SEMICYUC registry (Spanish Working Group on Severe Pandemic Influenza A (GETGAG) of the Spanish Society of Critical Care Medicine and Coronary Units (SEMICYUC)). All patients with influenza symptoms admitted to the participating ICUs were tested for influenza A or B, and investigators voluntarily registered all influenza A (H1N1)pdm09-positive patients in the national registry. The identification of patients was anonymized and individual patient informed consent was not obtained given the noninterventional nature of the study. The GETGAG/SEMICYUC registry was approved by the Institutional Review Board of Hospital Joan XXIII University Hospital of Tarragona, Spain.

All patients admitted to the ICU with clinical manifestations of respiratory infection in which influenza A (H1N1)pdm09 virus was identified during the first week of hospital stay were included in the study. The presence of influenza A (H1N1)pdm09 virus was confirmed by real-time polymerase chain reaction (rt-PCR) performed according to recommendations of the Centers for Disease Control and Prevention (CDC) [24]. Clinical manifestations included two or more of the following signs and symptoms: fever (>38 °C), cough, bronchial expectoration, and myalgias associated with clinical signs of organ or system failure (respiratory failure, hemodynamic instability, renal failure, or altered consciousness). Exclusion criteria were patients younger than 15 years of age, patients diagnosed with influenza A (H3N2) or influenza B, and patients in whom diagnosis of influenza A (H1N1)pdm09 virus infection had been established from 7 days of hospital admission.

### Definitions

Patients included in the study were classified into two groups according to the time at which the diagnosis of influenza A infection was made: early (within the first 2 days of hospital admission) and late (between the 3rd and 7th day of hospital admission). Definition of community-acquired pneumonia was based on recommendations of the American Thoracic Society/Infectious Diseases Society of America (ATS/IDSA) [25].

### Case report form

A case report form (CRF) was designed for data collection, including demographics (age, sex), time-related variables (time between hospital admission and diagnosis of influenza A, length of hospital stay before ICU admission, length of ICU stay, total length of hospital stay), comorbidities, previous influenza vaccination, epidemics season (2009–2012, 2013–2015), severity of illness, presenting manifestations of infection (pneumonia, severe asthma, acute exacerbation episode of a chronic pulmonary disease, heart failure), treatments administered (antivirals, inotropic drugs, corticoids, mechanical ventilation, extrarenal depuration procedures), and intra-ICU mortality. The severity of infection was assessed according to the Acute Physiology and Chronic Health Evaluation (APACHE II) score [26] and the Sequential Organ Failure Assessment (SOFA) score [27] on ICU admission. Information was provided by physicians of the participating ICUs according to the patient’s medical history, laboratory data, and radiological findings. The predicted mortality (based on APACHE II score) in the early and late diagnosis groups versus the observed mortality was calculated using the online APACHE II calculator (http://clincalc.com/IcuMortality/APACHEII.aspx).

### Statistical analysis

Categorical variables are expressed as frequencies and percentages, and continuous variables as mean and standard deviation (SD) when data followed a normal distribution or as median and interquartile range (25th–75th percentile) when the distribution departed from normality. Differences between groups were analyzed with the chi-square (χ^2^) test or the Fisher’s exact test for categorical variables, and the Student’s *t* test or the Mann-Whitney *U* test for continuous variables. Significant variables in the bivariate analysis were included in a multivariate logistic regression model to assess independent factors associated with late diagnosis and mortality. Odds ratios (ORs) and 95 % confidence intervals (CIs) were calculated. Cumulative survival for patients with influenza A (H1N1)pdm09 virus infection according to time of diagnosis was assessed using the Kaplan–Meier plot. Statistical significance was set at *P* < 0.05. Data were analyzed using the Statistical Package for the Social Sciences (SPSS, Chicago, IL, USA) for Windows 15.0.

## Results

**Fig. 1:**
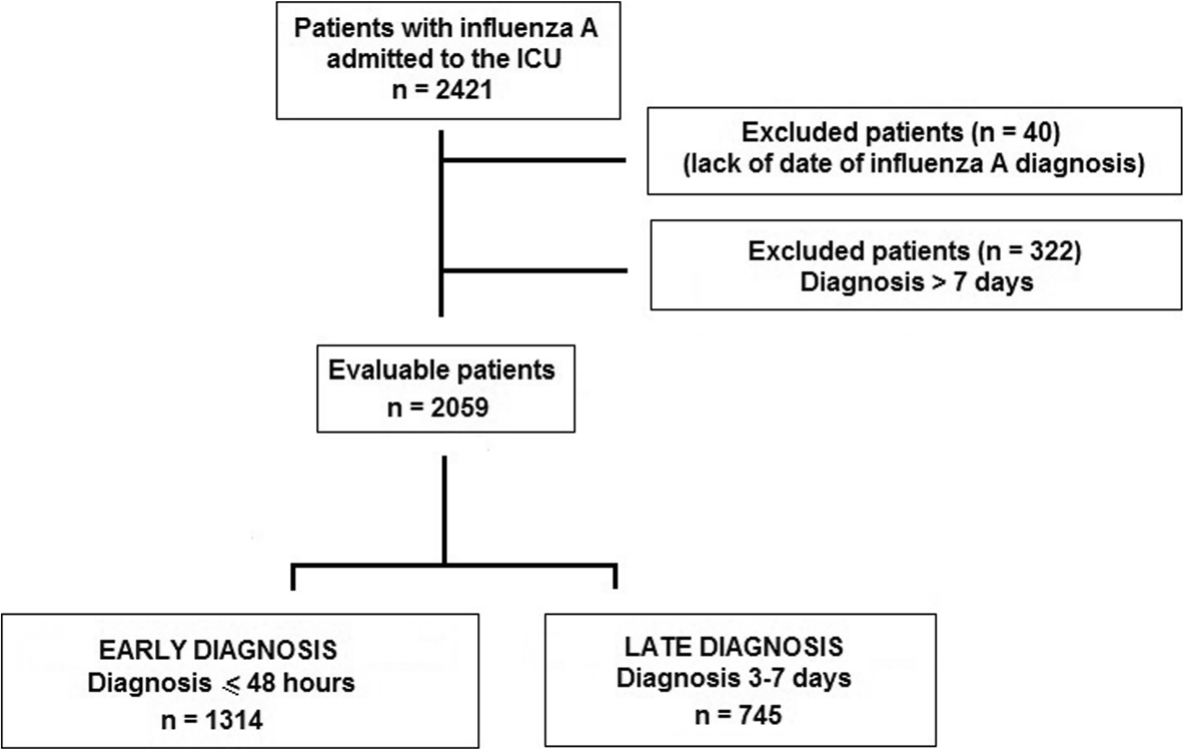
Distribution of patients with influenza A (H1N1)pdm09 virus infection admitted to the ICU according to the date of diagnosis

A total of 2421 patients diagnosed with influenza A (H1N1)pdm09 virus infection were included in the GETGAG/SEMICYUC registry. The diagnosis was established within the first week of hospital admission in 2059 (85.0 %) patients, 1314 (63.8 %) of whom were classified into the early diagnosis group and 745 (36.2 %) into the late diagnosis group (Fig. 1). Patients in the late diagnostic group, compared with those in the early diagnosis group, were significantly older, showed higher severity of illness, higher percentages of immunosuppression, hematological diseases, and chronic renal failure, required longer hospital and ICU stay, required invasive and noninvasive mechanical ventilation more frequently, required use of vasoactive drugs, corticoids, and extrarenal depuration procedures, and treatment with oseltamivir was prescribed more lately (Table 1).

**Table 1:** Descriptive characteristics of patients admitted to the ICU with early or late diagnosis of influenza A (H1N1)pdm09 virus infection and independent factors related to diagnostic delay

Variable	Early diagnosis (≤2 days)	Late diagnosis (3–7 days)	*P* value	Odds ratio (95 % confidence interval)	*P* value
Total patients	1314	745			
Age (years), mean (SD)	48.43 (15.6)	51.23 (15.0)	0.001	1.02 (1.01–1.03)	0.001
Sex					
Men	744 (56.6)	458 (61.4)	0.032		
Women	570 (43.4)	287 (38.5)			
Seasonal period					
2009–2012	732 (59.45	499 (40.5)	0.001	2.08 (1.64–2.63)	0.001
2013–2015	582 (70.3)	246 (29.7)			
Influenza vaccine	60 (4.6)	44 (5.9)	0.169		
Comorbid conditions					
Asthma	148 (11.3)	72 (9.7)	0.262		
Chronic obstructive pulmonary disease	235 (17.9)	159 (21.4)	0.054		
Heart failure	122 (9.3)	84 (11.3)	0.146		
Chronic renal failure	81 (6.2)	69 (9.2)	0.013		
Hematological disease	66 (5.0)	64 (8.6)	0.001		
Obesity	459 (34.9)	269 (36.1)	0.574		
Diabetes mellitus	189 (14.4)	116 (15.6)	0.432		
Human immunodeficiency virus infection	32 (2.4)	15 (2.0)	0.540		
Neuromuscular disease	39 (3.0)	16 (2.2)	0.269		
Autoimmune disease	41 (3.1)	28 (3.8)	0.381		
Immunosuppression	105 (8.0)	102 (13.7)	0.001		
Pregnancy	53 (4.0)	29 (3.9)	0.879		
APACHE II score, mean (SD)	15 (7)	16 (8)	0.001		
SOFA score, mean (SD)	6 (3)	6 (4)	0.001		
Presenting clinical manifestations					
Primary viral pneumonia	1129 (85.9)	603 (81.0)	0.004		
Coinfection (bacterial pneumonia)	219 (16.7)	133 (17.9)	0.458		
Noninvasive mechanical ventilation	482 (36.67	314 (42.2)	0.022		
Mechanical ventilation	864 (65.0)	569 (76.4)	0.001	1.58 (1.17–2.13)	0.002
Days on mechanical ventilation, median (IQR)	8 (2–15)	9 (4–20)	0.001		
Vasoactive drugs	639 (48.6)	425 (57.0)	0.001		
Decubitus prono	233 (17.7)	143 (19.52	0.382		
Continuous venovenous hemofiltration	103 (7.8)	95 (12.8)	0.001	1.54 (1.08–2.18)	0.016
Corticoids	527 (40.1)	335 (45.0)	0.043		
Days on corticoids, median (IQR)	7 (4–10)	7 (5–12)	0.071		
Days until ICU admission, median (IQR)	1 (1–1)	1 (1–3)	0.001	1.26 (1.17–1.35)	0.001
Length of ICU stay (days), median (IQR)	8 (4–17)	10 (5–20)	0.001		
Length of hospital stay (days), median (IQR)	14 (8–25)	18 (10–30)	0.001		
Days until oseltamivir therapy, median (IQR)	4 (2–6)	5 (3–7)	0.001		
Mortality rate	225 (17.1)	200 (26.9)	0.001		

Data expressed as frequencies (percentages) unless otherwise stated

*APACHE* Acute Physiology and Chronic Health Evaluation, *IQR* interquartile range (25th–75th percentile), *SD* standard deviation, *SOFA* Sepsis-related Organ Failure Assessment

In the logistic regression analysis, independent variables related to a delay in diagnosis of influenza A (H1N1)pdm09 virus infection were as follows: age (OR = 1.02, 95 % CI 1.01–1.03, *P* < 0.001); first seasonal epidemics (2009–2012) (OR = 2.08, 95 % CI 1.64–2.63, *P* < 0.001); stay of in-patient care before ICU admission (OR = 1.26, 95 % CI 1.17–1.35, *P* < 0.001); and need for mechanical ventilation (OR = 1.58, 95 % CI 1.17–2.13, *P* = 0.002) and continuous venovenous hemofiltration (OR = 1.54, 95 % CI 1.08–2.18, *P* = 0.016) (Table 1). Patients admitted to the ICU within the first 48 hours of hospitalization showed a mean (SD) APACHE II score of 15 (7) vs 18 (8) for patients admitted after the first 48 hours (*P* < 0.001). Also the mortality rate was significantly different between ICU admission within 48 hours of hospitalization and after 48 hours (19.4 % vs 35.2 %, *P* < 0.001).

**Fig. 2:**
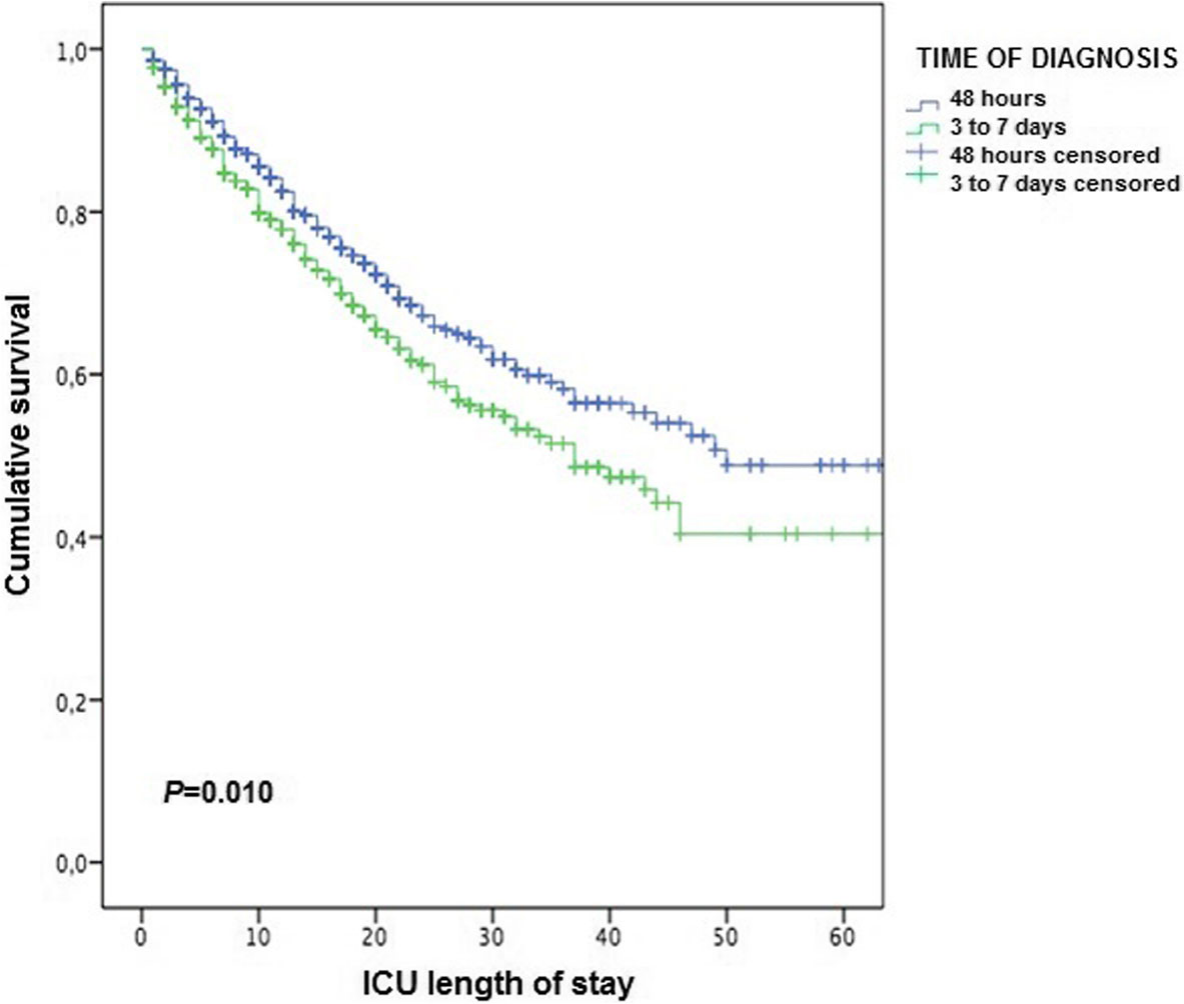
Kaplan–Meier survival curves for critically ill patients admitted to the ICU with confirmed influenza A (H1N1)pdm09 in the early and late diagnostic groups

**Fig. 3:**
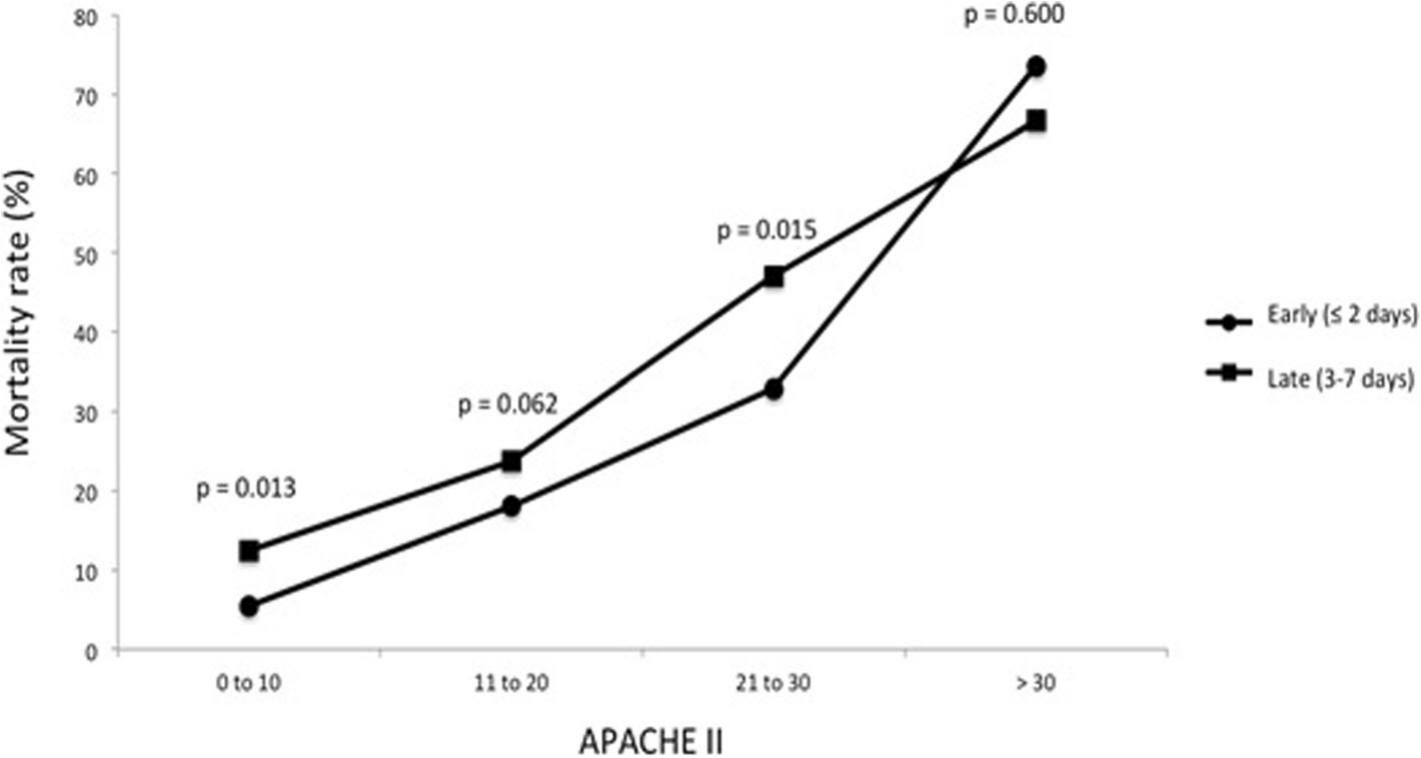
Relationship between severity of illness on ICU admission (APACHE II score) and mortality in the early and late diagnosis of influenza A (H1N1)pdm09 virus infection. *APACHE* Acute Physiology and Chronic Health Evaluation

The intra-ICU mortality was 17.1 % in the early diagnosis group (predicted 22 %) and 26.9 % in the late diagnosis group (predicted 23.5 %) (*P* < 0.001). Time to event analysis showed an association between timing of influenza A (H1N1)pdm09 diagnosis and mortality (Fig. 2), although in both groups mortality was related to the severity level (APACHE II score) on ICU admission (Fig. 3). Independent of the severity level on admission, mortality was significantly higher in the late diagnostic group for APACHE II scores of 0–10 and 21–30. Statistical significance was almost reached for APACHE II score of 11–20 (*P* = 0.062) and was not significant for scores > 30 (Fig. 3). A further subanalysis regarding delay in oseltamivir therapy in relation to the date on which influenza A infection was diagnosed showed no significant differences in mortality (≤1 day vs > 1 day, 17.9 % vs 22.8 %, *P* = 0.153; ≤ 2 days vs > 2 days, 19.0 % vs 23.3 %, *P* = 0.085; ≤ 3 days vs > 3 days, 20.6 % vs 23.2 %, *P* = 0.222). In relation to immunosuppression, the mortality rate was higher in the group of late diagnosis of influenza A (H1N1)pdm09 virus infection than in the early diagnosis group both in the presence of immunosuppression (55.2 % vs 40.6 %, *P* = 0.046) and in the absence of immunosuppression (22.7 % vs 14.9 %, *P* = 0.001).

As shown in Table 2, independent factors significantly associated with intra-ICU mortality in patients diagnosed with influenza A (H1N1)pdm09 virus infection within the first week of hospital admission included the following: late diagnosis (OR = 1.36, 95 % CI 1.03–1.81, *P* < 0.001); APACHE II score on ICU admission (OR = 1.09, 95 % CI 1.07–1.11, *P* < 0.001); hematological disease (OR = 1.98, 95 % CI 1.23–3.19, *P* < 0.001); need for mechanical ventilation (OR = 4.84, 95 % CI 2.73–8.56, *P* < 0.001); and use of continuous venovenous hemofiltration (OR = 4.81, 95 % CI 3.31–7.01, *P* < 0.001).

**Table 2:** Patients diagnosed with influenza A (H1N1)pdm09 virus: differences between survivors and patients who died, and independent factors related to mortality

Variable	Survivors	Patients who died	*P* value	Odds ratio (95 % confidence interval)	*P* value
Total patients	1528	395			
Age (years), mean (SD)	48.32 (15.24)	53.08 (15.51)	0.001		
Sex					
Men	865 (56.6)	255 (64.6)	0.004		
Women	662 (43.4)	140 (35.4)			
Seasonal period					
2009–2012	941 (80.7)	225 (19.3)	0.053		
2013–2015	587 (77.5)	170 (22.5)			
Influenza vaccine	65 (4.3)	26 (6.6)	0.137		
Comorbid conditions					
Asthma	113 (11.6)	18 (6.6)	0.019		
Chronic obstructive pulmonary disease	291 (19.0)	73 (18.5)	0.824		
Heart failure	140 (9.2))	53 (13.4)	0.011		
Chronic renal failure	82 (5.4)	53 (13.4)	0.001		
Hematological disease	65 (4.3)	52 (13.2)	0.001	1.98 (1.23–3.19)	0.001
Obesity	523 (34.2)	139 (35.2)	0.683		
Diabetes mellitus	212 (13.9)	65 (16.5)	0.183		
Human immunodeficiency virus infection	26 (1.7)	17 (4.3)	0.002		
Neuromuscular disease	44 (2.9)	11 (2.8)	0.929		
Autoimmune disease	45 (2.9)	20 (5.1)	0.036		
Immunosuppression	99 (6.5)	89 (22.5)	0.001		
Pregnancy	64(4.2)	11 (2.8)	0.203		
APACHE II score, mean (SD)	14 (6)	21 (8)	0.001	1.09 (1.07–1.11)	0.001
SOFA score, mean (SD)	5 (3)	8 (4)	0.001		
Presenting clinical manifestations					
Primary viral pneumonia	1273 (83.3)	342 (86.6)	0.105		
Coinfection (bacterial pneumonia)	229 (15.0)	96 (24.3)	0.001		
Noninvasive mechanical ventilation	527 (34.75	141 (35.7)	0.330		
Mechanical ventilation	812 (53.1)	366 (92.7)	0.001	4.84 (2.73–8.56)	0.001
Vasoactive drugs	664 (43.5)	307 (77.7)	0.001		
Decubitus prono	206 (13.05	133 (33.7)	0.001		
Continuous venovenous hemofiltration	67 (4.4)	116 (29.4)	0.001	4.81 (3.31–7.01)	0.001
Corticoids	500 (32.7)	196 (49.6)	0,001		
Days until ICU admission, median (IQR)	1 (1–2)	1 (1–3)	0.001	1.05 (0.99–1.11)	0.117
Length of ICU stay (days), median (IQR)	8 (4–18)	9 (4–18)	0.660		
Length of hospital stay (days), median (IQR)	16 (10–30)	11 (5–21)	0.001		
Days until oseltamivir therapy, median (IQR)	4 (2–6)	5 (3–6)	0.054		
Time of diagnosis					
Early (≤2 days)	1035 (67.7)	214 (54.2)	0.001	1.36 (1.03–1.81)	0.001
Late (3–7 days)	493 (32.3)	181 (45.8)			

Data expressed as frequencies (percentages) unless otherwise stated

*APACHE* Acute Physiology and Chronic Health Evaluation, *IQR* interquartile range (25th–75th percentile), *SD* standard deviation, *SOFA* Sepsis-related Organ Failure Assessment

## Discussion

This study shows that diagnostic delay of community-acquired influenza A (H1N1)pdm09 virus infection in critically ill patients admitted to the ICU is a risk factor for mortality. Late versus early diagnosis of influenza was associated with more days of hospitalization before ICU admission, greater need for respiratory support and extrarenal depuration techniques, as well as longer durations of stay in the ICU and in the hospital.

The selection of 7 days as a time limit for considering the community setting as the source of influenza A (H1N1)pdm09 virus infection is based on the limit established for the incubation period of the virus [28]. The incubation period estimated for the healthy population ranges between 2 and 4 days [29, 30], although in adult patients and in immunosuppressed patients a more prolonged period has been described [31]. The present study therefore considered that the origin of infection was the community for all patients with compatible symptoms of respiratory tract infection in whom a definitive diagnosis of influenza A (H1N1)pdm09 was made within the first week of hospital admission, whereas the origin was probably nosocomial when diagnosis was established from the second week of hospital admission.

Although the study was not designed to assess causes of delay in diagnosis of influenza A (H1N1)pdm09 virus infection (specific reasons were not included in the registry), it is likely that late diagnosis may be related to the lack of clinical suspicion of viral infection or to negative results in the respiratory samples initially analyzed. The first case usually corresponds to patients with suspicion of bacterial infections treated empirically with antimicrobials with poor clinical response, and the second case to difficulties in obtaining and/or processing adequate samples. In the first publications of patients admitted to the ICU with influenza A (H1N1)pdm09 virus infection during the 2009 H1N1 influenza pandemic, upper respiratory samples were negative in up to 20 % of cases, so the definitive diagnosis could have been established in samples recovered from the lower respiratory tract [7, 8]. Obtaining new samples from bronchial aspirates is thus recommended for patients with suspected severe viral pneumonia and negative oropharyngeal samples, and bronchoalveolar lavage samples should be collected only if results of bronchial aspirates are persistently negative [32].

In our country, we found a decrease in the number of patients with late diagnosis during the second influenza epidemic season, which may be due to a training effect in the management of patients with clinical suspicion of influenza A (H1N1)pdm09 virus infection especially during outbreaks and due to greater availability of techniques for rapid diagnosis.

In the present study, clinical characteristics associated with diagnostic delay of influenza A (N1H1)pdm09 were examined. Although clinically relevant differences between patients in the early and late diagnosis groups were found for a number of variables in the univariate analysis, only age, seasonal period, mechanical ventilation, continuous venovenous hemofiltration, and days until ICU admission were predictors of diagnostic delay in the logistic regression analysis. In our study there was a quite long interval between the day of blood sampling and the onset of treatment with oseltamivir even in the early diagnostic group, which may indicate that in most cases treatment was not started until the physician in charge was aware of positivity of influenza A (H1N1)pdm09 testing. Other reasons for late diagnosis, such as low degree of vigilance or false negative tests, were not recorded. Also, it has been shown that patients admitted to the ICU within the first 48 hours of hospitalization had a significantly lower severity level and mortality than those admitted to the ICU after 48 hours of hospitalization. According to these findings, a high level of clinical suspicion of influenza A (H1N1)pdm09 infection in patients at risk during flu outbreaks is needed, to establish the diagnosis as soon as possible and to reduce both delayed admission to the ICU and specific treatment with oseltamivir. Patients at risk include nonvaccinated patients (which in our study have been most of the patients in both groups) and patients in whom vaccination is recommended.

The overall intra-ICU mortality was significantly higher in the late diagnosis group. The predicted mortality based on APACHE II score on ICU admission was higher (22 %) than the observed mortality (17.1 %) in the early diagnosis group, but lower in the late diagnosis group (23.5 % vs 26.9 %). The reason why delay in diagnosis of influenza A (H1N1)pdm09 virus infection is associated with worse outcome is unclear, and a number of factors including a difference in days until oseltamivir therapy, delay in ICU admission, high severity of illness, some comorbidities, or other unidentified variables could have played a complementary role. Patients in the late diagnosis group showed higher APACHE II and SOFA scores on ICU admission, but data for severity of illness on hospital admission were not recorded, so it is unknown whether patients were already more severe on hospital admission or worsened during hospitalization due to lack of an early diagnosis, appropriate treatment, or prompt ICU admission. Up to the present time, a number of factors related with mortality in patients diagnosed with influenza A (H1N1)pdm09 virus infection have been reported, including age, severity of illness on admission, underlying immunosuppression, delay in starting specific antiviral treatment (oseltamivir), duration of symptoms before the initiation of treatment, presence of hematological or cardiac disease, need for mechanical ventilation or extrarenal depuration techniques, and dyspnea or signs of alteration of the central nervous system on admission, among others. The present study provides the first observation that a delay in the diagnosis of influenza A (H1N1)pdm09 may be an independent factor associated with a higher mortality rate. Our data are complementary to other observations in which mortality is related with a delay in starting antiviral treatment and a greater duration of clinical signs of infection prior to diagnosis of infection by influenza A (H1N1)pdm09 virus [20]. For this reason, in critically ill patients admitted to the ICU and because of the increase in mortality associated with a delay in diagnosis, it is recommended to initiate antiviral treatment on diagnostic suspicion [32, 33].

Some limitations of the study should be taken into account. Firstly, the classification used for defining early and late diagnosis is based on epidemiological and microbiological considerations (viral shedding time) upon which there is no consensus in the literature. The selection of 48 hours was arbitrary. Considering that the criterion to define the time of diagnosis of influenza A was the day of blood sampling that allowed the identification of infection, and because this technique is not available in the emergency laboratories of some Spanish hospitals, 48 hours was assumed as the cutoff point given that in many centers a sample for PCR assay was electively collected on the next day of admission to the emergency department. However, a further analysis with early diagnosis at ≤24 hours and late diagnosis at 2–7 days showed similar results (data not shown). Differences in clinical characteristics and outcome between patients in the early and late diagnostic groups emphasize the need for including this classification to homogenize risk groups in future studies. On the other hand, retrospective analysis of an epidemiological prospective database prevents the inclusion of new variables that might have been of help to define the proposed classification. The multicenter design of the study in which a therapeutic protocol has not been established previously may be associated with treatment bias, given that treatments considered most adequate were those used by each participating group. Moreover, certain variability in the interpretation of clinical signs might be present, although consensuated definitions were used for most study variables.

## Conclusions

This study shows important differences in patients diagnosed with influenza A (H1N1)pdm09 virus infection depending on the speed with which the infection is diagnosed. Late diagnosis of community-acquired influenza A (H1N1)pdm09 infection is associated with a higher severity of illness, delay in ICU admission, need for therapeutic resources, greater duration of ICU and hospital stay, and, more importantly, higher intra-ICU mortality. The present findings highlight the need during the epidemiological seasons for an early diagnosis of influenza A (H1N1)pdm09 and prompt antiviral treatment in all hospitalized patients with signs of respiratory infection, independently of other clinical diagnoses.

## Abbreviations

APACHE II: Acute Physiology and Chronic Health Evaluation
CDC: Centers for Disease Control and Prevention
CI: Confidence interval
GETGAG: Spanish Working Group on Severe Pandemic Influenza A
HIV: Human immunodeficiency virus
ICU: Intensive care unit
OR: Odds ratio
rt-PCR: Real-time polymerase chain reaction
SEMICYUC: Spanish Society of Critical Care Medicine and Coronary Units
SOFA: Sepsis-related Organ Failure Assessment
